# Liver function maximum capacity test during normothermic regional perfusion predicts graft function after transplantation

**DOI:** 10.1007/s13167-024-00371-7

**Published:** 2024-07-16

**Authors:** Ivo J. Schurink, Femke H. C. de Goeij, Fenna J. van der Heijden, Rutger M. van Rooden, Madeleine C. van Dijk, Wojciech G. Polak, Luc J. W. van der Laan, Volkert A. L. Huurman, Jeroen de Jonge

**Affiliations:** 1 Division of HPB and Transplant Surgery, Department of Surgery, Erasmus MC Transplant Institute, Erasmus University Medical Center, Doctor Molewaterplein 40, 3015 GD Rotterdam, Zuid Holland The Netherlands; 2 LUMC Transplant Center, Department of Surgery, Leiden University Medical Center, Leiden, The Netherlands

**Keywords:** Liver transplantation, Donation after circulatory death, Normothermic regional perfusion, Liver function maximum capacity (LiMAx), Viability assessment, Prognostic assessment, Predictive preventive personalized medicine (PPPM)

## Abstract

**Purpose:**

In an effort to reduce waitlist mortality, extended criteria donor organs, including those from donation after circulatory death (DCD), are being used with increasing frequency. These donors carry an increased risk for postoperative complications, and balancing donor-recipient risks is currently based on generalized nomograms. Abdominal normothermic regional perfusion (aNRP) enables individual evaluation of DCD organs, but a gold standard to determine suitability for transplantation is lacking. This study aimed to incorporate individualized and predictive measurements of the liver maximum capacity (LiMAx) test to objectively grade liver function during aNRP and prevent post-op complications.

**Methods:**

aNRP was performed to salvage 18 DCD liver grafts, otherwise discarded. Continuous variables were presented as the median with the interquartile range.

**Results:**

The liver function maximum capacity (LiMAx) test was successfully performed within the aNRP circuit in 17 aNRPs (94%). Donor livers with good lactate clearance during aNRP demonstrated significantly higher LiMAx scores (396 (301–451) µg/kg/h versus those who did not 105 (70–158) µg/kg/h; *P* = 0.006). This was also true for manifesting stress hyperglycemia > 20 mmol/l (*P* = 0.032). LiMAx score correlated with alanine aminotransferase (ALT; *R* =  − 0.755) and aspartate transaminase (AST; *R* =  − 0.800) levels during perfusion and distinguished livers that were selected for transplantation (397 (346–453) µg/kg/h) from those who were discarded (155 (87–206) µg/kg/h; *P* < 0.001). Twelve livers were accepted for transplantation, blinded for LiMAx results, and all had LiMAx scores of > 241 µg/kg/h. Postoperatively, LiMAx during aNRP displayed correlation with 24-h lactate levels.

**Conclusions:**

This study shows for the first time the feasibility to assess liver function during aNRP in individual donor livers. LiMAx presents an objective tool to predict donor liver function and risk of complications in the recipient, thus enabling individualized matching of donor livers for an individual recipient. The LiMAx test may present a valuable test for the prediction of donor liver function, preventing post-transplant complication, and personalizing the selection of donor livers for individual recipients.

**Supplementary Information:**

The online version contains supplementary material available at 10.1007/s13167-024-00371-7.

## Introduction

### Organ shortage in liver transplantation

Liver transplantation is the only curative treatment for end-stage liver diseases. In Europe, nearly 8000 liver transplantations from deceased donors are performed annually [1]. Due to persisting organ shortage, many patients die on the waiting list or are removed because of (oncological) disease progression. In an attempt to alleviate the shortage, regular acceptance criteria are stretched to expand the donor pool. These extended criteria donor (ECD) grafts bear donor risk factors such as moderate to severe steatosis, worsening laboratory values before donation, or arise from donation after circulatory death (DCD). DCD grafts are more prone to postoperatieve complications, compared to donation after brain death (DBD) grafts. Especially primary non-function (PNF), early allograft dysfunction (EAD), non-anastomotic biliary strictures (NAS), and acute kidney injury are more frequent [2, 3]. A key factor contributing to this increased risk is additional injury to the graft during the agonal phase. The impact of the functional warm ischemia time (fWIT) during the agonal phase is a black box, of which the outcome is unpredictable and may vary from hardly any clinical relevance to PNF [4]. The lack of currently available tools to predict post-transplantation outcomes drives transplant surgeons to generally be reluctant to accept DCD livers with any additional risk factors. The organ utilization rate for DCD grafts is thus largely reduced, compared to DBD grafts [5]. In order to overcome the discard of potentially usable DCD grafts, a more personalized approach is warranted to predict the individual liver function capacity of the donor liver, predict the outcome of transplantation of a specific liver graft, and to potentially apply preventive strategies in particular grafts to mitigate the risk of a complicated post-transplantation course.

### Predictive, Preventive, and Personalized Medicine in liver transplantation

Predictive, Preventive, and Personalized Medicine (PPPM) represents a proactive healthcare paradigm, using innovative biotechnologies to refine disease prediction, strengthen preventive strategies, and customize therapeutic approaches [6]. While widely studied and applied in diseases such as diabetes mellitus and various cancers, the PPPM principles have not yet been integrated in liver transplantation, although careful consideration of multifaceted donor organ quality is vital to determine the outcome for individual recipients. Currently, balancing donor liver risk factors to recipient risks is done based on generalized nomograms, arising from risk factors for graft failure. The Balance of Risk (BAR) score and the United Kingdom (UK) DCD donor-recipient risk index take, e.g., into account donor and recipient factors, such as age donor BMI, ischemic time in the donor, predicted cold ischemia time, recipient sickness at time of transplant, and retransplantation status [7, 8]. Based on points scored on these items, futility to use an individual liver graft for a specific recipient is determined, without actually testing the function or viability of that individual donor organ. This highlights the significance to have predictive tools to determine individual donor organ function to predict postoperative function and prevent complication, so that an individualized donor/recipient match can be made.

### Donor liver evaluation during abdominal normothermic regional perfusion

The incorporation of organ perfusion techniques, such as abdominal normothermic regional perfusion (aNRP), during the procurement phase of transplantation has introduced novel instruments to comply with the PPPM strategy. aNRP uses extracorporeal membrane oxygenation in the abdominal compartment of the donor after the agonal phase and declaration of death to stop ischemic injury, resuscitate ischemic organs, and replenishes energy levels. It also enables the unique possibility to assess individual donor liver function and the result of incurring additional liver injury during the dying process of the donor (donor functional warm ischemia; fWIT) [9]. More and more centers from Europe as well as in the United States of America are implementing aNRP in their standard practice [10–14]. The post-transplantation results of aNRP grafts are excellent, and the complication rate resembles that of DBD grafts. Notably, the incidence of NAS is low with 0–10% [11–14], compared to 10–30% in non-treated DCD grafts [13, 15, 16]. The assessment of the liver during aNRP to determine suitability for transplantation is however subjective. In a recent systematic review [17], the authors have demonstrated that grafts are predominantly evaluated on macroscopic aspect. The macroscopic aspect was the reason for decline in 48% of the cases. Lactate clearance was incidentally used as a function marker. The drawback of lactate measurement during aNRP is that lactate levels are influenced by anoxic blood leaking from the periphery back to the circuit, or by the administration of lactate-rich fluids, such as Ringer’s lactate and packed red blood cells [12]. Consequently, the absence of an objective and reliable liver function test within current aNRP protocols highlights a significant gap and does not align with the PPPM framework [6, 17]. Hence, there is a relatively high percentage of non-accepted donor livers (29%), indicating that underutilization of liver grafts remains an issue. This underutilization is further compounded by stringent inclusion criteria to initiate aNRP, driven by caution due to the unfeasibility of analyzing true liver function during aNRP.

### Innovative approach for assessing liver function

Along the PPPM strategy, routine implementation of a real-time test, truly reflecting individual liver function, would be an example of promoting predictive diagnostics to prevent complications in the recipient, while at the same time maximizing donor organ utilization. This test should be feasible during aNRP, a procedure targeted to prevent failure of the hepatocyte and cholangiocyte compartment. Based upon the results of such a test, individual decisions to accept a specific liver for a specific recipient can be taken, minimizing organ discard and at the same time reducing post-transplant morbidity for recipients.

An optimal function test would (1) exclusively measure liver function, (2) be comparable between procedures, and (3) not be influenced by other factors that can differ between aNRP procedures. A substrate-based specific liver function test exposes the donor liver to an equivalent dosage of substrate which is metabolized exclusively by the liver, indicating liver function. The liver function maximum capacity (LiMAx) test is a potential candidate test that analyzes the conversion of the substrate Methacetin via the cytochrome P450 1A2 (CYP1a2) system [18]. In this clinically approved breath test, Methacetin is labelled with a heavier ^13^C isotope that is converted by CYP1a2 into paracetamol and ^13^CO_2_ [18]. The produced ^13^CO_2_ is measured in exhaled air and is compared to ^12^CO_2_. We recently showed that ^13^CO_2_ also can be detected in “exhaled” gas in an oxygenator during organ machine perfusion [19]. A LiMAx score can then be calculated from the ratio of ^13^CO_2_:^12^CO_2_, which is demonstrated to have a predictive value in chronic and acute liver diseases [20–23].

## Working hypothesis and aims

As mentioned above, there is a lack of real-time individual liver function testing during aNRP in liver transplantation, presenting a critical gap to predict outcome in current procedures. A promising method to address this gap is the use of the LiMAx test. Our study has two primary objectives: first, to adapt and optimize the LiMAx test for utilization during aNRP, and second, to investigate its potential association with aNRP parameters and subsequent post-transplantation outcomes. By exploring this novel application of the LiMAx test, our research aims to advance the PPPM paradigm within liver transplantation. By refining the selection process for donor livers and optimize machine perfusion strategies, we strive to improve the outcome of individual donor organ – patient combinations, and alleviate the burden of organ shortage on transplant waiting lists.

## Materials and methods

All patients undergoing liver transplantation with an aNRP DCD donor liver between February 1, 2020, and February 28, 2022, were analyzed. All research was conducted in accordance with both the Declarations of Helsinki and Istanbul. Patients provided informed consent for the use of outcome data and the protocol was approved by the medical ethical committee of the Erasmus Medical Center (MEC 2019–0370).

### Abdominal normothermic regional perfusion

aNRP was performed using the Donor Assist (Xvivo, Groningen, the Netherlands) as previously described [13]. Briefly, the organ retrieval procedures were performed by specialist stand-alone organ procurement teams of the Erasmus Medical Center and the Leiden University Medical Center in DCD donors (Maastricht III & V), which were deemed unsuitable for transplantation by all Euro-transplant centers in the normal allocation procedure. The abdominal aorta and inferior caval vein were cannulated and the proximal aorta was cross-clamped just above or below the diaphragm. Subsequently, aNRP was initiated. The target flow was > 1.7 l/min and the target arterial oxygen partial pressure (pO2) was between 100 and 200 mmHg. During the study period, the acceptance criteria were altered, allowing more hepatocyte injury and less biliary injury. At the start, the liver was considered for transplantation if alanine aminotransferase (ALT) in the perfusate was stable and < 200U/l, and the lactate level was decreasing. Sufficient bile quality was required, defined as a pH > 7.45 and glucose < 3.0 mmol/l. In September 2021, the acceptance criteria were changed towards ALT in the blood being stable and < 400U/l, lactate level decreasing at least 1 mmol/h and the liver producing > 4 ml bile. Sufficient bile quality was defined as glucose < 3.0 mmol/l, and additionally delta bicarbonate bile vs. perfusate > 5 and delta pH bile vs. perfusate > 0.1.

### LiMAx test

**Fig. 1:**
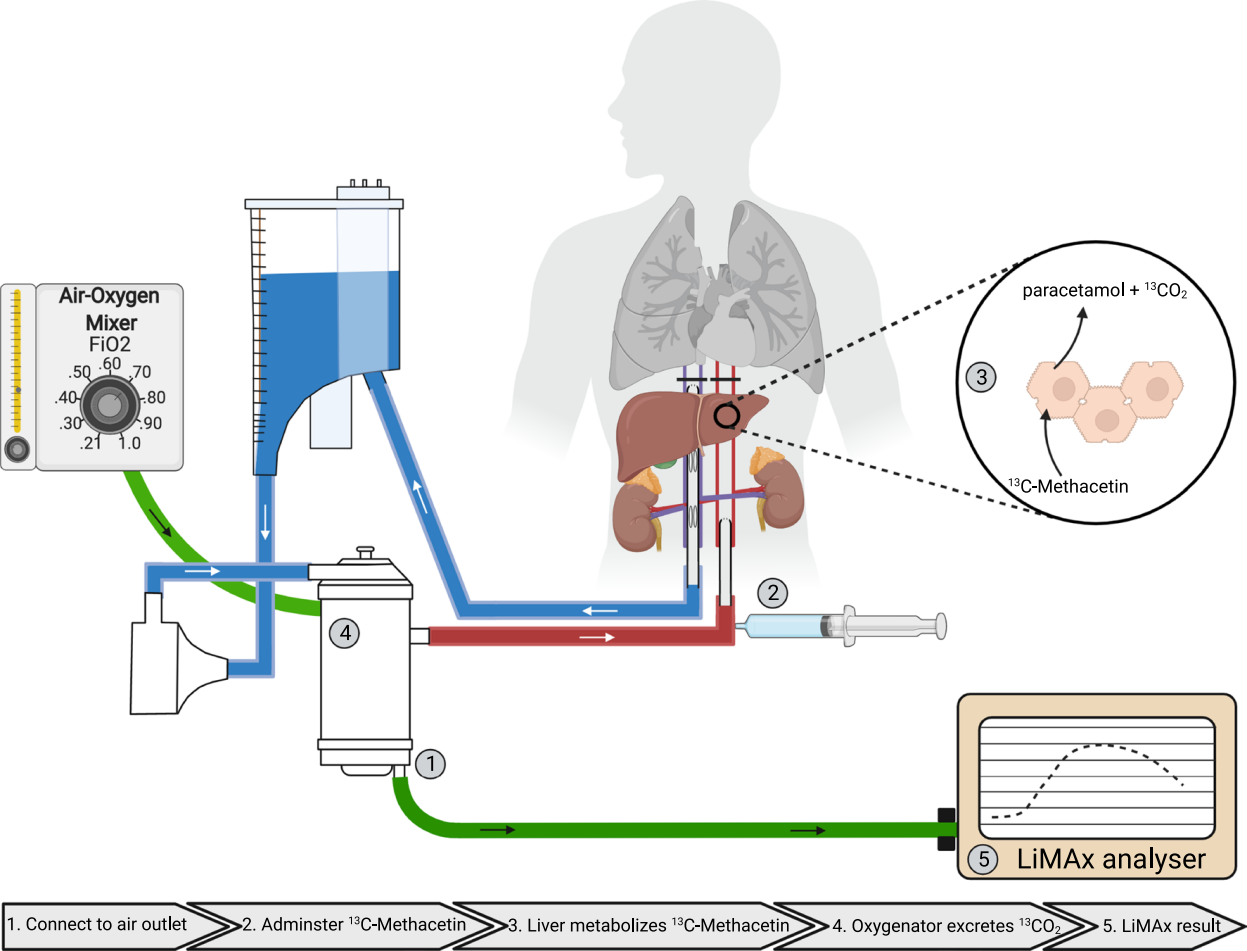
Graphic summary of LiMAx testing during the aNRP procedure. (1) The LiMAx analyzer is connected to the air outlet of the membrane oxygenator and the baseline ^13^CO_2_:^12^CO_2_ ratio is measured. (2) The ^13^C-methacetin is administered after 60 min of aNRP. (3) The ^13^C-methacetin is metabolized in the hepatocytes into ^13^CO_2_ and paracetamol. 4. The ^13^CO_2_ is released from the perfusate into the “exhaled” air from the oxygenator. (5) The LiMAx analyzer analyses the “exhaled” air and produces a curve illustrating delta over baseline (DoB) against the time in minutes. In addition to measuring DoB in exhaled air, total CO_2_ production is also assessed. The LiMAx score is then calculated based on both DOB_max_ and total CO_2_ production

The A2D Analyzer (ArgosMED GmbH, Karlsruhe, Germany) was connected to the gas outlet of the oxygenator of the aNRP disposable circuit (Fig. 1). Five minutes before administering the ^13^C-Methacetin, a baseline ratio of 13C labeled carbon dioxide (^13^CO_2_): 12C labeled carbon dioxide (^12^CO_2_) was recorded. The dosage of ^13^C-Methacetin (Humedics GmbH, Berlin, Germany) was 2 mg per kg donor body weight. After 60 min of aNRP, ^13^C-Methacetin was administered as a bolus to the arterial inflow line, followed by 20 ml of NaCl 0.9%. The duration of the LiMAx test was set at a maximum of 60 min. The changes in the ratio of ^13^CO_2_:^12^CO_2_ are presented as delta over baseline (DoB). The LiMAx score was calculated according to the following formula [19]:

$$\text{LiMAx}=\frac{{DoB}_{\text{max}}\times \text{C}\times \text{P}\times \text{ M}}{\frac{1}{2}D}$$

The LiMAx score is presented in µg/kg/h. DoB_max_ implies the maximum recorded DoB value. “C” presents a constant (*C* = 0.011237). “P” presents the production of CO_2_ (mmol/hour). “M” represents the molar mass of ^13^C-Methacetin (*M* = 166.19 g/mol). “D” is the dosage of ^13^C-Methacetin in milligrams. In the original formula by Stockmannet al. [18], CO_2_ production (P) was estimated as the average CO_2_ production per body surface. In our series, we precisely measured the CO_2_ production via analysis of the carbon dioxide partial pressure (pCO_2_) in the outgoing air from the oxygenator with a handheld capnograph (Microcap™; Ordion Medical; Jerusalem; Israel). The pCO_2_ was converted into CO_2_ production based on the general gas equation [24].

### Liver transplantation

Liver transplantation was performed using the standard caval vein-sparing technique with side-to side anastomosis. Since September 2021, all DCD livers underwent mandatory dual hypothermic machine perfusion (DHOPE) perfusion, according to the protocol of van Rijn et al. [25]. In the earlier period, some grafts underwent DHOPE perfusion for logistic reasons.

### Outcomes and definitions

The graft and recipient characteristics were collected. Patient and graft survival at 90 days were calculated. Graft survival was defined as the duration from the initial liver transplantation until either re-transplantation or patient death. All relevant outcome measures including PNF, post-reperfusion syndrome, and postoperative EAD score were collected. PNF was defined as early allograft failure resulting in either recipient death or re-transplantation within 72 h postoperatively, in the absence of any vascular complication. Post-reperfusion syndrome was defined as a decrease of more than 30% in the mean systemic arterial blood pressure within 10 min after reperfusion [26]. The following risk scores were used: Eurotransplant donor risk index (ET-DRI), UK DCD Risk Score, and EAD following the Olthoff criteria [7, 27, 28]. NAS was defined as clinical symptoms in combination with radiologically proven non-anastomotic biliary strictures. The start of fWIT was defined as the time at which the donor oxygen saturation is < 80% and/or the mean arterial blood pressure is < 50 mmHg. The asystolic time is defined as the time between the circulatory arrest and the initiation of aNRP. The analyses of the liver biopsies are described in the supplementary data.

### Statistical analysis

Categorical variables were presented as the number and the percentage. Continuous variables were presented as the median with the interquartile range. Comparisons between groups were done for categorical variables with the chi-square or Fisher’s exact test and for the continuous variables the Mann–Whitney-*U* test was used. Normal distribution was tested with a Kolmogorov-Smirnov test. Correlations between two variables were calculated with a Pearson correlation coefficient. Tests were considered statistically significant if a two-sided *P* value was < 0.05. Statistical analyses were performed with IBM SPSS Statistics 25 (IBM Corp. Released 2017. IBM SPSS Statistics for Windows, Version 25.0. Armonk, USA).

## Results

**Fig. 2:**
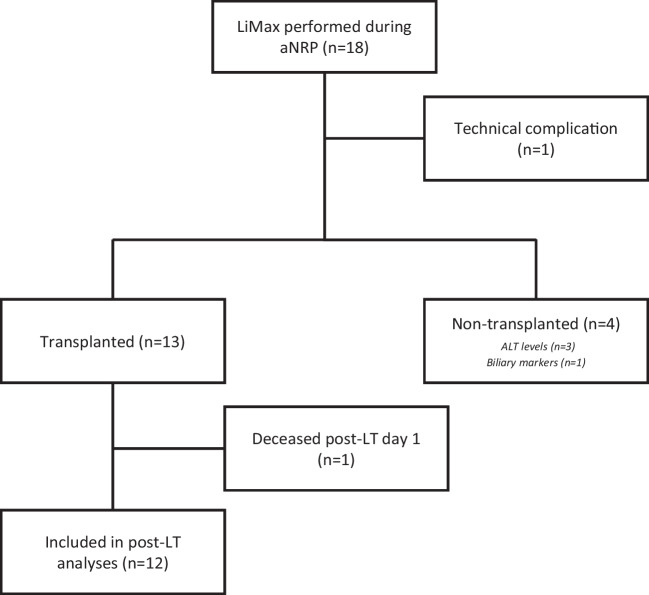
Flowchart of donor livers for this study. LiMAx was performed in 18 aNRP procedures. During one attempt, a technical failure occurred. From the remaining 17 aNRP procedures, 13 livers were transplanted, while 4 livers were declined based on pre-defined criteria

Between February 2020 and February 2022, 18 extended DCD donors were offered to the Dutch aNRP program, in which a LiMAx test was performed (Fig. 2). Of these 18 procedures, one LiMAx test failed due to a gas leak in the connection tube from the oxygenator to the A2D laser system, affecting the LiMAx result. In the other 17 procedures, the LiMAx test was successful. From these 17 liver grafts, 13 grafts passed the acceptance criteria, as described in the method section, and were transplanted. The other four grafts failed the acceptance criteria and were therefore declined for transplantation, because of ALT levels outside protocol in three cases and insufficient biliary quality in one case (Table 1).

**Table 1:** Donor and NRP characteristics of transplanted and non-transplanted livers

	Transplanted (*n* = 13)	Non-transplanted (*n* = 4)	*P*
Age	69 (65–71)	69 (68–70)	0.799
BMI	26 (23–29)	24 (23–24)	0.426
Sex (male)	6 (46%)	1 (25%)	0.603
Cause of death			0.723
* Trauma*	2 (15%)	1 (25%)	
* Cerebrovascular attack*	10 (77%)	2 (50%)	
* Anoxia*	0 (0%)	0 (0%)	
* Other*	1 (8%)	1 (25%)	
fWIT (minute)	33 (29–35)	49 (46–51)	**0.017**
Asystolic time	22 (19–24)	24 (20–31)	0.649
Laboratory values before withdrawal of life-sustaining therapy			
AST (U/l)	34 (25–47)	48 (38–122)	0.125
ALT (U/l)	30 (21–39)	58 (38–116)	0.063
GGT (U/l)	25 (18–51)	22 (15–33)	0.572
ALP (U/l)	67 (55–73)	72 (64–76)	0.703
NRP characteristics			
Perfusion time (minutes)	112 (108–123)	99 (92–108)	0.106
Perfusion characteristics at end of NRP			
Flow (l/m)	2.2 (1.9–2.4)	1.9 (1.8–2)	0.156
ALT (U/l)	40 (30–70)	428 (294–546)	**0.005**
AST (U/l)	50 (43–98)	425 (310–524)	**0.001**
pH perfusate	7.27 (7.20–7.30)	7.19 (7.13–7.22)	0.258
Bicarbonate perfusate	15.1 (13.6–16.9)	13.5 (10.6–15.2)	0.195
Lactate perfusate	10.3 (8.9–12.0)	17.1 (14.8–19.0)	**0.014**
Glucose perfusate	16.3 (11.3–17.2)	14.2 (12.8–17.4)	0.849
Production of bile	12 (100%)	3 (75%)	0.235
Total bile production (ml)	22 (14–30)	5 (2–9)	0.013
pH bile	7.66 (7.57–7.70)	7.36 (7.34–7.56)	0.521
Bicarbonate bile	25.2 (23.3–31.7)	17 (15.9–18.5)	0.057
Glucose bile	1.0 (1.0–1.0)	2.2 (1.6–4.3)	**0.029**

Significant *P* values are emphasized in bold

Donor, donation, and aNRP characteristics are described in Table 1. Out of all donation characteristics, only the fWIT was significantly different between the transplanted group and non-transplanted group (33 (29–35) min versus 49 (46–51) min; *P* = 0.017). During aNRP, the ALT and aspartate transaminase (AST) levels were significantly lower in the transplanted group (ALT: 40 (30–70)U/l; AST: 50 (43–98)U/l) compared to the non-transplanted group (ALT: 428 (294–546)U/l; *P* = 0.005; AST: 425 (310–524)U/l; *P* = 0.001; Supplementary Fig. 1). Lactate levels at the end of aNRP were significantly lower in the transplanted group (10.3 (8.9–12.0) mmol/l) than in the non-transplanted group (17.1 (12.8–17.4 mmol/l; *P* = 0.014). Also, cumulative bile production was significantly higher in the transplanted group (22 (14–30) ml) compared to the non-transplanted group (5 (2–9) ml; *P* = 0.013).

### Recipients and transplantation characteristics

Recipient characteristics and transplant results of the 13 transplanted livers are described in Table 2. One out of 13 patients (8%) suffered from a post-reperfusion syndrome. Lactate levels 24 h after transplantation were normalized, except for one patient with portal vein thrombosis. The median lactate value was 1.2 (1.1–1.4) mmol/l. The median international normalized ratio (INR) 24 h after transplantation was 1.4 (1.3–1.7). The median ALT and AST levels 24 h after transplantation were 350 (224–481)U/L and 462 (276–560)U/l (Table 2; Supplementary Fig. 2). Two patients (15%) experienced early allograft dysfunction, according to Olthoff’s definition. Graft survival at 6-months and 12-months were both 92%. None of the patients suffered from PNF or hepatic artery thrombosis. One recipient, suffering from extensive porto-mesenteric thrombosis before transplantation, deceased in the early postoperative period due to recurrent mesenteric thrombosis not related to the liver graft quality. No incidence of NAS was observed.

**Table 2:** Donor and recipient characteristics and postoperative results

	NRP (*n* = 13)
Donation characteristics	
Age	69 (65–71)
Donor hepatectomy time (minutes)	23 (19–30)
ET-DRI	2.99 (1.68–3.18)
UK-Risk index	11 (8–14)
Recipient characteristics	
Age	64 (50–67)
Sex (male)	11 (85%)
BMI	28 (25–30)
Lab Meld-score	9 (7–12)
Transplantation indication	
-HCC	8 (62%)
-Cirrhosis	1 (8%)
-Biliary disease	2 (15%)
-Other	2 (15%)
Transplantation	
DHOPE performed	5 (38%)
Static cold storage (minutes)	295 (265–345)
Anastomosis time (minutes)	27 (25–31)
Estimated blood loss (ml)	3000 (1900–8000)
Packed red blood cells (ml)	540 (0–1080)
Fresh frozen plasma (ml)	700 (0–1800)
Operation time (minutes)	364 (329–417)
24-h post-operative laboratory results	
Lactate (mmol/l)	1.2 (1.1–1.4)
ALT (U/l)	350 (224–481)
AST U/l)	462 (276–560)
LDH (U/l)	324 (249–423)
Bilirubin (mmol/l)	18 (13–41)
INR	1.4 (1.3–1.7)
Post-operative complications	
Post-reperfusion syndrome	1 (8%)
Primary non-function	0 (0%)
EAD	2 (15%)
Hepatic artery thrombosis	0 (0%)
NAS	0 (0%)
Portal vein thrombosis	1 (8%)
Graft survival 6 month	92%
Graft survival 12 month	92%

### LiMAx during aNRP

**Fig. 3:**
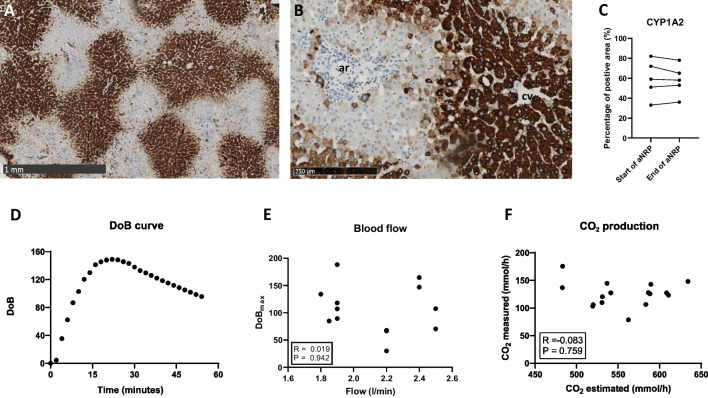
Histologic evaluation of biopsies from aNRP perfused donor livers and technical aspects of the LiMAx test. **A** and **B** demonstrate the staining of liver biopsies with CYP1A2 immunohistochemistry. **A** consists of a low magnification image of 30 × , demonstrating that CYP1A2 is expressed in the hepatocytes in metabolic zone 2 (mid-lobular) and 3 (pericentral). **B** consists of a high magnification image of 100 × , demonstrating that the CYP1A2 enzyme is present in the cytoplasm of the hepatocytes. **C** demonstrates the percentage of the area that is positive on the CYP1A2 staining in the biopsies taken at the start of aNRP and at the end of aNRP in 5 donor livers. **D** shows an example of a typical LiMAx curve during aNRP. **E** demonstrates independence of the DOBmax from the blood flow during aNRP. **F** demonstrates the correlation between the measured total CO2 production and the estimated total CO2 production

First, we determined the anatomical location of the CYP1A2 enzyme which is responsible for the metabolism of ^13^C-methacetin into paracetemol and ^13^CO_2_. The CYP1A2 enzyme was only present in the hepatocytes (Fig. 3A, B). The expression of the enzyme across hepatocytes depends on the metabolic zonation; we found the CYP1A2 enzyme to be predominantly present in pericentral hepatocytes (metabolic zone 3) and mid-lobular hepatocytes (metabolic zone 2). In the hepatocyte, the CYP1A2 enzyme was intensely present in the cytoplasm of the cells (Fig. 3B). The area of the biopsies that was positive for CYP1A2 staining was at the start of aNRP 59 (51–72)%, and the end of aNRP 58 (53–68)% (Fig. 3C). The presence of CYP1A2 enzyme did not change during aNRP.

The LiMAx test was performed in the donor after 60 min of aNRP. Figure 3D demonstrates a typical DoB curve. It took between 2 and 6 min until the first signal was detected. The DoB_max_ was reached between 11 and 29 min. The height of the DoB_max_ was flow-independent (*R* = 0.019; Fig. 3E). The DoB_max_ values ranged between the 17 and 188 and presented the ratio of ^13^CO_2_:^12^CO_2_. To convert the DoB into a LiMAx score, it is pivotal to correct for total CO_2_ production, which in the original publication is estimated during LiMAx measurement in a patient [18]. During aNRP, the estimated CO_2_ production of the donor was 567.1 (531.2–589.7) mmol/h, while the actual measured total CO_2_ production during aNRP was however 127.6 (120.0–142.9) mmol/h. No correlation was found between the estimated total CO_2_ production and the actual measured total CO_2_ production (*R* =  − 0.083; *P* = 0.759); Fig. 3F). The resulting LiMAx score of all aNRP procedures ranged between 35 and 510 µg/kg/h. No significant correlations were identified between the LiMAx score and donor factors, such as donor age, donor BMI, or the last donor AST and ALT before withdrawal of life-sustaining treatment (Supplementary Fig. 3A–D). Similarly, no correlation was found between the LiMAx score and donor fWIT or asystolic time (Supplementary Fig. 3E-F).

**Fig. 4:**
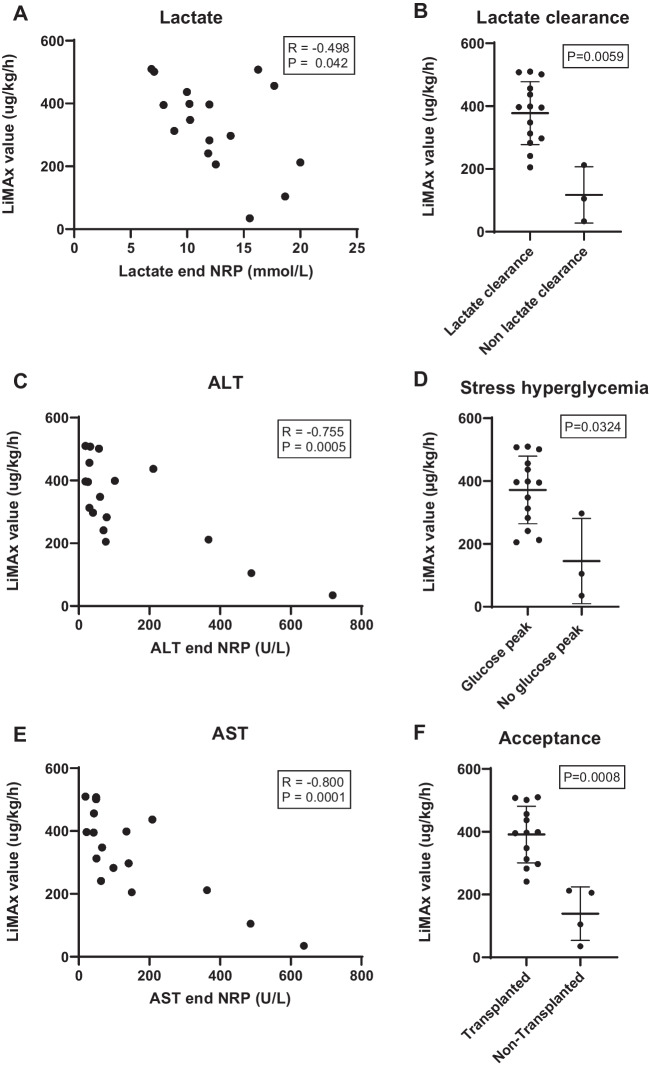
The LiMAx score during aNRP is compared to liver parameters (Lactate, ALT, AST, and glucose peak) of aNRP. **A** shows significant correlation between LiMAx score and lactate levels in the blood at the end of aNRP. **B** shows the LiMAx score according to dichotomous lactate clearance during aNRP. Donor livers which demonstrated non lactate clearance had a significant lower LiMAx score. **C** and **E** demonstrate significant correlation between LiMAx scores and ALT and AST levels in the blood at the end of aNRP. **D** demonstrates that the livers with a stress glucose peak (> 20 mmol/l) have a higher LiMAx score. **F** shows that transplanted livers had a higher LiMAx score than non-transplanted livers

Comparing the LiMAx scores to aNRP parameters, a significant negative correlation was found between the LiMAx scores and the lactate levels at the end of aNRP (*R* =  − 0.498; *P* = 0.0420; Fig. 4A). Donor livers which cleared lactate during aNRP had a higher LiMAx score (396 (301–396) µg/kg/h) than livers which did not: (105 (70–158) µg/kg/h; *P* = 0.0059; Fig. 4B). Likewise, donor livers that showed a stress hyperglycemia peak > 20 mmol/l, had a higher LiMAx score (396 (290–456) µg/kg/h), compared to livers that had a lower glucose peak (105 (70–201); *P* = 0.0324; Fig. 4D). No significant correlation was seen between LiMAx score and bile production (Supplementary Fig. 4). We did however find a strong negative correlation between the LiMAx score and the measured hepatocellular damage markers (AST: *R* =  − 0.800; *P* = 0.0001, ALT: *R* =  − 0.755; *P* = 0.0005; Fig. 4C and E).

**Fig. 5:**
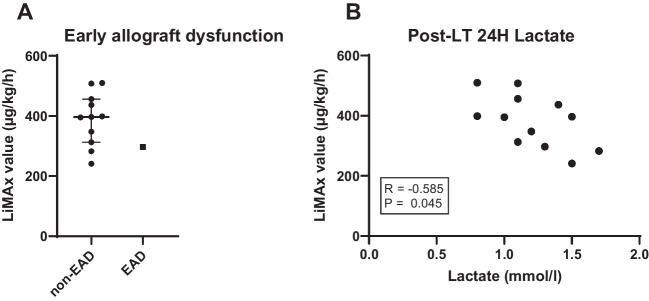
The LiMAx score compared to the post-transplantation results. **A** shows the donor aNRP LiMAx score compared to early allograft dysfunction in the first week after transplantation. **B** shows LiMAx score correlated to lactate levels at 24 h post-transplantation

The LiMAx scores of the 13 transplanted livers were significantly higher (397 (313–453) µg/kg/h), compared to the 4 non-transplanted livers (155 (87–207) µg/kg/h; *P* = 0.0008; Fig. 4F). All transplanted livers had a LiMAx score above 241 µg/kg/h during aNRP. The patient who suffered from recurrent mesenteric vein thrombosis shortly after reperfusion had a LiMAx score of 501 µg/kg/h. This patient was excluded from the analyses of the LiMAx scores in relation to post-transplantation outcome. Out of the remaining 12 liver grafts, 4 (33%) had a minimal amount of steatosis (> 5%), 7 grafts had moderate steatosis (5–30%), and 1 graft had severe steatosis (50%). The LiMAx score of the minimally steatotic grafts was 354 (309–396) µg/kg/h, that of the moderately steatotic grafts was 372 (299–427) µg/kg/h and in the severely steatotic graft the score was 507 µg/kg/h. No difference was seen between the minimal and moderate steatotic grafts (*P* = 0.648). Furthermore, no difference in LiMAx score was noted in the livers undergoing additional DHOPE or not. Post transplantation, one of the grafts had EAD with a LiMAx score of 297 µg/kg/h, while the other liver grafts had a median LiMAx score of 397 (330–447) µg/kg/h (Fig. 5A). All lactate levels at 24 h post-transplantation were within a normal range, but still showed a significant negative correlation to the LiMAx score (*R* =  − 0.585; *P* = 0.045; Fig. 5B). Other laboratory values at 24 h did not show a correlation with the LiMAx score. ALT level was 427 (271–538) U/l, AST level was 342 (214–397) U/l, lactate dehydrogenase (LDH) level was 309 (241–362) U/l and INR was 1.4 (1.3–1.7) at 24 h after reperfusion.

## Discussion

Our results demonstrate that the implementation of a clinically validated, substrate-based specific liver function test, is feasible during aNRP. We show that the LiMAx score is both correlated to liver function markers and liver injury markers during aNRP. Furthermore, we demonstrated that transplanted extended criteria DCD donor grafts with a LiMAx score above 241 µg/kg/h all functioned well post-transplantation.

During aNRP, high-risk donor livers are evaluated by the donor surgeon in order to prevent serious post-transplant complications. Currently, liver graft acceptance criteria during aNRP predominately exist in the macroscopic aspect (eye of the surgeon), with additional more objective criteria such as a low amount of microscopic steatosis and fibrosis, ALT levels lower than 200 U/l, and a downward lactate trend [17]. This conservative policy results in excellent post-transplantation outcomes in controlled DCD grafts; however, at the cost of a rather low organ utility rate. In contrary to aNRP for uncontrolled DCD grafts, where the current acceptance criteria overestimate the predicted outcome of donor liver, resulting in a high incidence of PNF (6–8%) [29, 30]. This mismatch can be overcome if aNRP assessment would be extended with a reliable biomarker of true liver function, independent of confounding issues during perfusion. The LiMAx test might be an excellent candidate test to realize this.

### Technical aspects of LiMAx in aNRP

The LiMAx breath test is based on the conversion rate of CYP1a2. This enzyme is exclusively expressed in hepatocytes [31], making the enzymatic conversion of the ^13^C-methacetin into ^13^CO_2_ liver-specific and thus a surrogate marker of global hepatocellular liver function. It is not affected by other abdominal organs that are included in the aNRP circuit. The benefit of the LiMAx test over the use of lactate trend as a marker for liver function is that all liver grafts are exposed to an equivalent amount of ^13^C-methacetin, which makes the LiMAx result comparable between different liver grafts. Furthermore, ^13^C-methacetin is an exogenous substrate, and all produced ^13^CO_2_ is picked up by the device. As a result, neither anoxic blood leaking from non-perfused parts to the perfusion circuit nor hemodilution during the aNRP procedure interferes with the LiMAx results, as opposed to traditional serum markers. The drawback of the LiMAx test includes susceptibility to produced ^12^CO_2_, e.g., due to changes in perfusion flow. Additionally, the administration of packed red blood cells, ringer lactate, or bicarbonate, temporarily affects the total CO_2_ concentration and will thus influence the DoB. Therefore, during the measurement (about 10 min from administration to peak DoB), it is preferred to have a stable perfusion. To optimize the LiMAx test during aNRP, we measured CO_2_ production instead of estimating this via the method originally described by Stockmannet al*.* [18]. As mentioned before, correct measurement of CO_2_ is important, as the LiMAx analyzer determines a ratio of ^13^CO_2_:^12^CO_2_. When the total CO_2_, which predominately consists of ^12^CO_2_, is not measured correctly, the DoB is not accurately converted into a LiMAx score, resulting in either an over- or an underestimation of the liver function. The formula for estimating the total CO_2_ production is designed for patients, and not for aNRP, in which only the abdominal compartment produces CO_2_ (bowels, kidney, liver, pancreas, etc.). We demonstrated that there was no relationship between estimated CO_2_ production in a patient and actual CO_2_ production in the abdominal compartment during perfusion. This highlights the importance of actually measuring the total CO_2_ production, which can be easily done using a handheld clinical anesthesia capnograph.

### Predictive value of LiMAx

The LiMAx score, reflecting hepatocellular function, should primarily protect from primary non-function. In this light, it is important to realize that Hessheimer et al. demonstrated that the length of cold ischemia time after aNRP still is a risk factor affecting graft survival, as is redo-transplantation with an aNRP liver graft, compared to primary transplantation [14]. In our series, with extended DCD donor livers, all transplanted liver grafts had a LiMAx score above 241 µg/kg/h. These grafts had excellent immediate post-transplant function. Lactate levels normalized within the first day and peak ALT levels were comparable or even better than comparative aNRP cohorts from literature (633–897 U/l) [12, 32, 33]. The ability of the LiMAx test to predict favorable biliary outcome and to protect from biliary complications, such as NAS, is however unknown. So far, no biliary complications were present in our group, with a median follow-up of 18 months. This might suggest that a LiMAx score > 241 µg/kg/h identifies extended DCD donor livers that will have good hepatocellular and cholangiocellular function after transplantation and can be safely used. Interestingly, no correlation was found between the amount of steatosis in the donor graft and the LiMAx score, indicating that individual steatotic extended DCD livers can have good metabolic function and that a tailor-made decision should be applied to maximize donor organ utilization.

### Optimal timing for the LiMAx test

Determination of the optimal timing to perform the LiMAx test remains an open question. In our previous study [34], we described the dynamic nature of liver function during machine perfusion and emphasized the importance of performing the LiMAx test at the same time point to be able to compare livers. In aNRP, routine perfusion is recommended for at least 1 h, up to a maximum of 4 h [35, 36]. As transplant clinicians know, it will take about an hour for a transplanted liver to start up metabolism, showing in bile production, increased clotting formation and pH regulation. To find an optimal balance between waiting for starting liver function, and swiftness to reach a decision on liver suitability to arrange recipient logistics, we chose to perform the LiMAx test 1 h after the start of aNRP. This timeframe aligns with a stable perfusion state and accommodates the varying durations of aNRPs, ranging from 73 to 133 min. Highlighting the cost aspect of the LiMAx test, the initial system purchase poses a substantial investment, approximately €50,000. However, the per-test expense stands at a reasonable €150 inclusive of all costs. We advocate for its strategic application in high-risk donor livers due to its promising cost-effectiveness—potentially preventing PNF and expanding the acceptance pool for viable livers.

### Limitations

The major limitation of this study is the small sample size and that not all liver grafts are transplanted. As this cohort comprises the first experience with aNRP in the Netherlands, non-use criteria were rather strict, and probably with increasing experience, more livers will be accepted for transplantation. The non-transplanted grafts were not used on our protocol-defined hepatocellular or biliary criteria, and all had a LiMAx score lower than 212 µg/kg/h. We will never know if PNF or other relevant complications would have occurred, but LiMAx scores lower than 100 µg/kg/h showed to be associated with increased mortality after major liver resection [37]. To gain more understanding of the role of LiMAx in predicting negative outcomes in liver transplantation, future research should aim to include a larger cohort with an adequate number of PNF cases and biliary complications.

## Conclusion and expert recommendations

In conclusion, we demonstrated that the LiMAx test is feasible during aNRP and it is the first objective liver-specific test to assess individual liver function during aNRP. At a level of 241 µg/kg/h, it predicts the safe use of extended DCD donor livers, without cases of PNF or NAS, and excellent graft survival. Yet, to determine the lower threshold until which donor organs can be accepted to maximize donor organ utilization, greater numbers need to be studied, including cases of graft failure.

### Predictive medical approach

Analyzing Methacetin metabolism through the CYP1A2 system test during aNRP can be considered the first objective liver-specific test to assess liver function in this context. Our findings contribute to introduce the predictive medical approach in liver transplantation by assessment of individual liver function during aNRP, instead of using subjective criteria or donor risk factors. The real-time data provided by the LiMAx test empowers clinicians to make informed decisions regarding the viability of individual donor livers and predict post-transplant outcomes, which apparently improves the quality of provided healthcare in comparison to the current liver transplantation procedures.

### Targeted prevention

The use of the LiMAx test during aNRP prevents catastrophic post-operative complications, such as primary non-function of the donor liver, or ischemia to the biliary tree, leading to cholangitis, biliary casts, and retransplanation. The LiMax test during aNRP allows clinicians to make informed decisions about the viability of the donor liver, and upon failure of the test, proactive regenerative medicine approaches can be taken to mitigate the risk of postoperative complications and therefore can be considered as secondary prevention [38, 39].

### Personalized treatments

The LiMAx test could signify a potential paradigm shift towards personalized/individualized treatment strategies in liver transplantation to optimize donor-recipient combinations. High-risk recipients, such as those with acute hepatic failure, portal vein thrombosis, or re-transplantation, frequently encounter poor outcomes post-transplantation [40–42]. Some of these indications also experience higher waitlist mortality rates [42]. Traditionally, these recipients have been limited to receiving grafts of optimal quality, capable to withstand prolonged cold ischemic times during expected difficulties in the recipient, and ensuring immediate graft function for the recipient in great need [41]. However, by assessing individual donor liver quality between the agonal phase and transplantation, the potential organ pool for these high-risk recipients would be largely expanded, potentially reducing morbidity and mortality in this vulnerable group.

Conversely, some low-risk recipients, which have to wait often longer due to their low Model for End-Stage Liver Disease status, may benefit from receiving a donor liver earlier. A recent study from Beumer et al. [43] revealed that recipients with an indication of hepatocellular carcinoma experienced a median survival decrease of 21% when they had to wait an additional 10 months for the liver transplantation. For these recipients, individual testing of presumably inferior donor livers, e.g. based on age, donor BMI, or worsening donor labs, could reduce the time on the waiting list and thus ultimately prove beneficial. The LiMAx test demonstrated to be safe to determine a safe threshold to pave the way for personalized treatment strategies, optimizing outcomes in liver transplantation.

### Contribution to a paradigm shift using PPM in liver transplantation

PPPM remains underexplored in the domain of liver transplantation, particularly in DCD liver transplantation. Implementing the PPPM framework in this context is inherently challenging due to the multifactorial nature of outcomes in (DCD) liver transplantation. Especially the donation phase introduces significant complexity to the procedure, rendering it non-straightforward. As aforementioned, the impact of fWIT during the agonal phase remains a “black box,” contributing to the unpredictability of DCD liver transplantation outcomes. This uncertainty places DCD liver transplantation within the realm of unPPPM (unpredictable, unpreventable, and impersonal medicine), as the outcomes cannot be reliably predicted or prevented using existing approaches [6]. Assessment of true liver function through the LiMAx test, which can be performed during aNRP, presents a promising opportunity to shift from reactive medicine to proactive, personalized approaches aligned with the principles of PPPM.

## Supplementary Information

Below is the link to the electronic supplementary material.

Supplementary file1 (DOCX 1465 KB)

## Data Availability

The data can be accessed upon reasonable request through the corresponding author.

## Abbreviations

ALP: Alkaline phosphatase
ALT: Alanine aminotransferase
aNRP: Abdominal normothermic regional perfusion
AST: Aspartate transaminase
BAR: Balance of Risk
BMI: Body mass index
^12^CO_2_: 12C labeled carbon dioxide
^13^CO_2_: 13C labeled carbon dioxide
CYP1a2: Cytochrome P450 1A2
DBD: Donation after brain death
DCD: Donation after circulatory death
DHOPE: Dual hypothermic machine perfusion
DoB: Delta over baseline
EAD: Early allograft dysfunction
ECD: Extended criteria donor(s)
ET-DRI: Eurotransplant donor risk index
(f)WIT: (Functional) warm ischemia time
GGT: Gamma glutamyltransferase
HAT: Hepatic artery thrombosis
ICU: Intensive care unit
INR: International normalized ratio
LDH: Lactate dehydrogenase
LiMAx: Liver function Maximum capacity
LT: Liver transplantation
NAS: Non-anastomotic biliary strictures
PNF: Primary non-function
pCO_2_: Carbon dioxide partial pressure
pO2: Oxygen partial pressure
PPPM: Predictive, Preventive, and Personalized Medicine
UK: United Kingdom
WLST: Withdrawal of life-sustaining treatment
