# Supplementary material for: Liver function maximum capacity test during normothermic regional perfusion predicts graft function after transplantation
Source: EPMA J. 2024 Jul 16;15(3):545–58. doi: 10.1007/s13167-024-00371-7 (PMC11372035; doi:10.1007/s13167-024-00371-7)
Supplement: Supplementary file 1 — Supplementary file1 (DOCX 1465 KB) [file 13167_2024_371_MOESM1_ESM.docx]

**Maximum liver function capacity test during abdominal normothermic regional perfusion as predictor of graft function after transplantation- Supplementary Results**

*Ivo J. Schurink, BSc, Femke H.C. de Goeij, BSc, Fenna J. van der Heijden, MD, Rutger M. van Rooden, MSc, Madeleine van Dijk, MSc, Wojciech G. Polak, MD, PhD, Luc J.W. van der Laan, PhD, Volkert A.L. Huurman, MD, PhD, Jeroen de Jonge, MD, PhD*


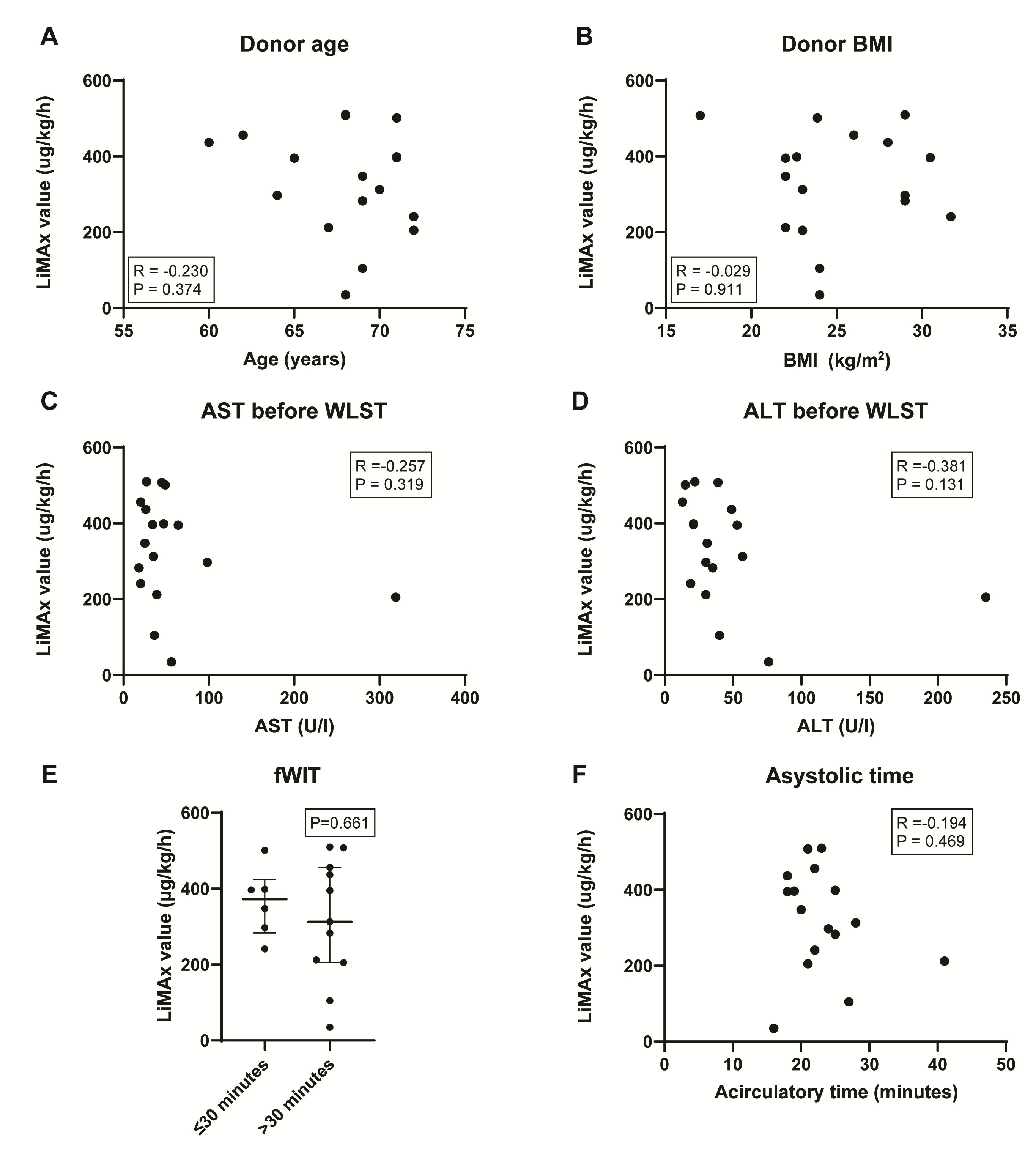


**Supplementary figure 1**: LiMAx score in relation to donor and donation characteristics before initiation aNRP. A. shows donor age. B. shows donor BMI. C. shows AST level before withdrawal of life-sustaining treatment. D. shows AST level before withdrawal of life-sustaining treatment. E. shows functional warm ischemia time. F shows asystolic time.

**Supplementary figure 2**: Flow chart of the acceptance parameters during aNRP. The blue line represents donor livers accepted for transplantation, while the red line represents donor livers declined based on pre-defined criteria during aNRP. Panels A to F depict various parameters: A. Lactate levels, B. Glucose levels, C. ALT levels, D. AST levels, E. Cumulative bile production, F. Blood flow.


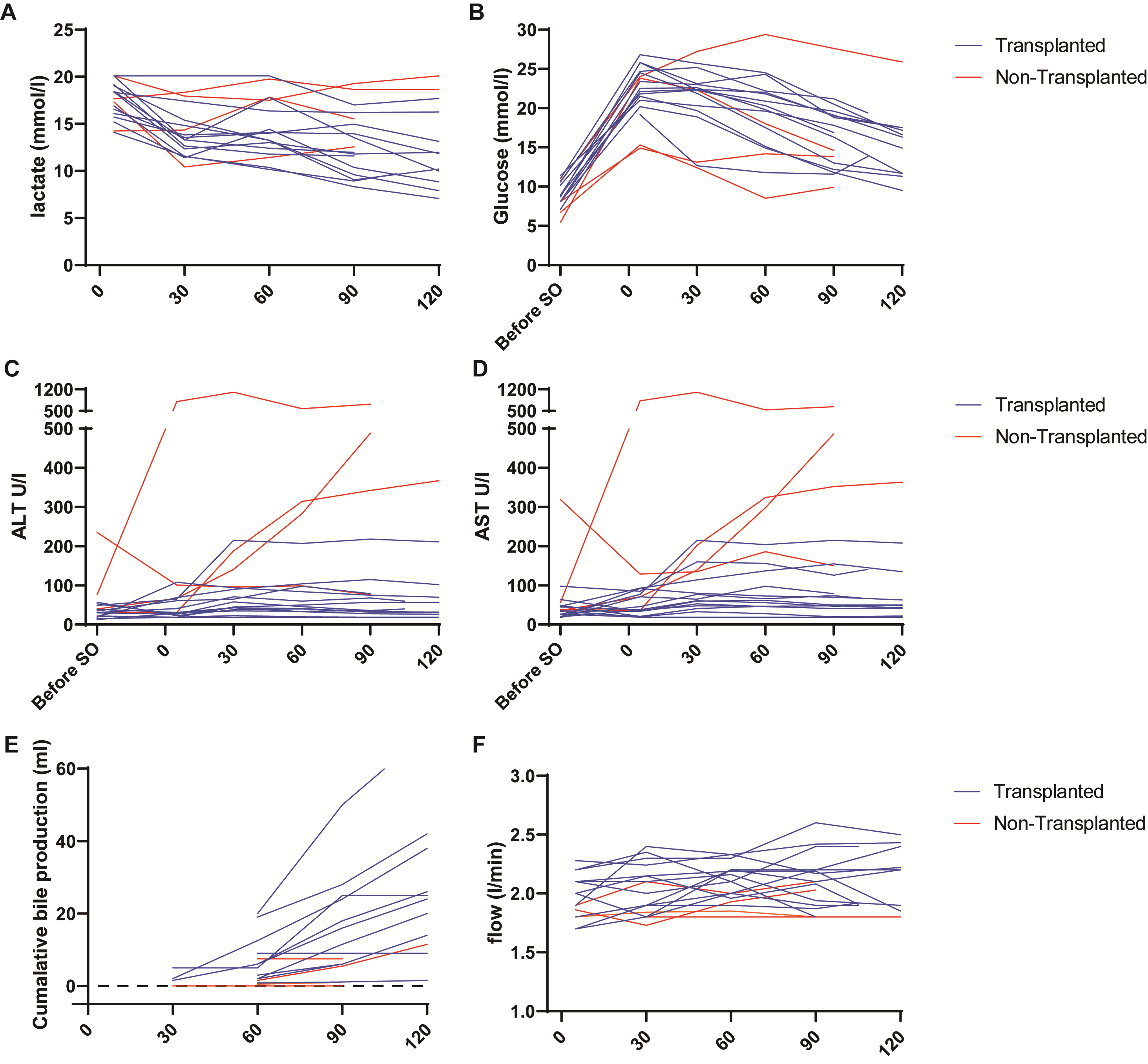


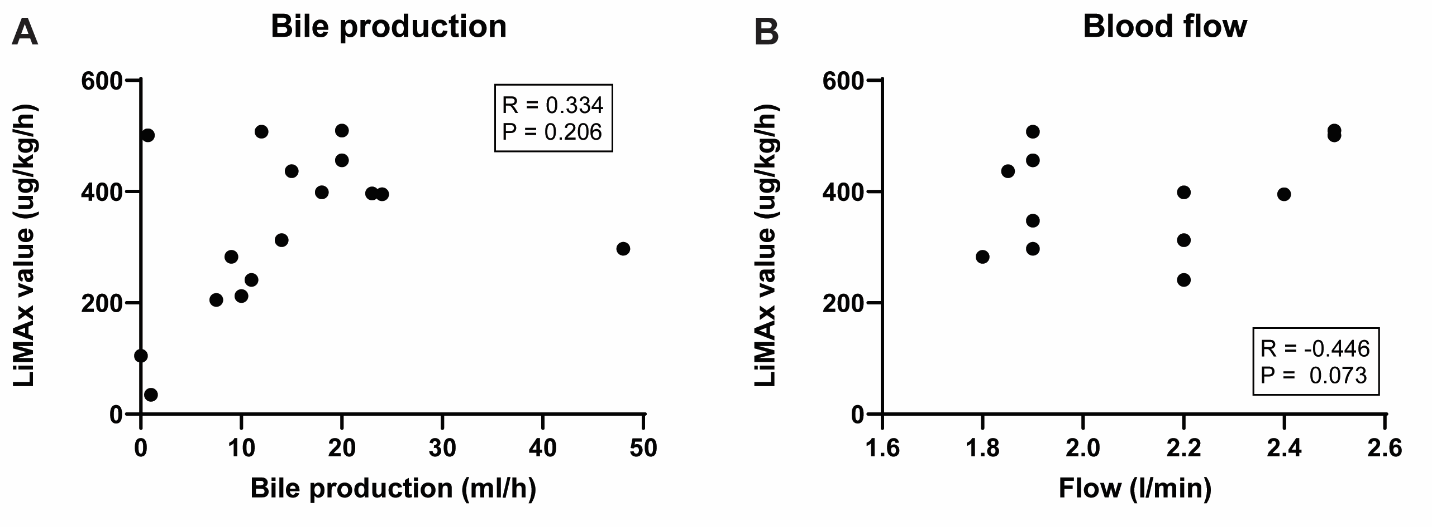


**Supplementary figure 3**: LiMAx score in relation to bile production during aNRP. No correlation was observed between the LiMAx score and bile production.
